# Innovation With People Living With Dementia: Creating New Narratives on Meaning, Belonging, Community, and Place

**DOI:** 10.1093/geroni/igaf122.974

**Published:** 2025-12-31

**Authors:** Fayron Epps, Jordan Lewis, Alison Phinney, Marigrace Becker

**Affiliations:** UT Health San Antonio, San Antonio, Texas, United States; Center on Aging, Thompson School of Social Work & Public Health, University of Hawai’i at Manoa, Honolulu, Hawaii, United States; The University of British Columbia, Vancouver, British Columbia, Canada; UW Medicine, Seattle, Washington, United States

## Abstract

This paper shares insights about innovation resulting from collaboration between dementia researchers and community-based organizations in North America, and how these collaborations can foster new ways of thinking and doing with people with dementia. The author focuses on examples from work with Black congregations to better support community members with dementia, with attention to institutional support for this type of collaboration, building trust with community partners, the role of effective marketing in extending impact beyond pilot projects, and the challenges of leading innovation.

